# Cold Snare Polypectomy for Large Sessile Colonic Polyps: A Single-Center Experience

**DOI:** 10.1155/2015/175959

**Published:** 2015-03-23

**Authors:** Thiruvengadam Muniraj, Ara Sahakian, Maria M. Ciarleglio, Yanhong Deng, Harry R. Aslanian

**Affiliations:** ^1^Section of Digestive Disease, Yale School of Medicine, New Haven, CT 06520, USA; ^2^University of Southern California, Los Angeles, CA 90089, USA; ^3^Department of Biostatistics, Yale Center for Analytical Sciences, Yale School of Public Health, New Haven, CT 06520, USA

## Abstract

Colonoscopic polypectomy has been shown to reduce the risk of colorectal cancer and the mortality. Postpolypectomy bleeding was reported to be lower with cold snare polypectomy (CSP) when compared with conventional polypectomy. CSP has traditionally been utilized only in smaller polyps below 1 cm. We retrospectively analyzed the CSP outcomes in patients with sessile polyps ≥10 mm in size and observed that CSP was feasible in large sessile polyps with no adverse events and with an acceptable rate of residual polyp on follow-up colonoscopy. Further prospective study in larger patient groups is warranted to determine optimal CSP techniques and whether CSP for large polyps has favorable efficacy in regard to complete polypectomy, procedure time, and complication rates relative to polypectomy with cautery.

## 1. Introduction

Colonoscopy has been shown to prevent incident colorectal cancers (CRC) with the identification and removal of polyps from the colon and rectum [1, 2]. Long-term results from a 30-year follow-up study of colonoscopy for patients at higher-than-average risk of colorectal cancer confirm that removing precancerous adenomas can not only reduce the risk of colorectal cancer but also reduce the mortality from the disease by more than half [3].

Sessile serrated polyps are frequently located in the proximal colon and are difficult to detect and remove completely with traditional polypectomy snares [4]. Inadequate identification and removal of sessile right colon polyps are suspected to be an important factor in studies which report reduced postcolonoscopy cancer reduction in the right versus left colon [5].

Polypectomy may be associated with complications in up to 10% of cases. Postpolypectomy bleeding occurs in approximately 1% of patients and polyp size is the major risk factor [6]. Several studies have shown that the cold snare polypectomy (CSP) technique is safe and effective and requires less time than the performance of snare-cautery [7]. Postpolypectomy bleeding [7] and abdominal symptoms were reported to be lower with CSP versus conventional polypectomy [8].

Prior studies, however, utilized CSP in small colorectal polyps, less than 1 cm in size and frequently less than 5 mm [9]. Animal study in a porcine model found CSP to be a safe and effective technique for flat colonic polyp removal up to 12 mm in size [10].

We report our experience and outcomes utilizing CSP for large/advanced sessile polyps greater than 10 mm in size.

## 2. Materials and Methods

**Figure 1:**
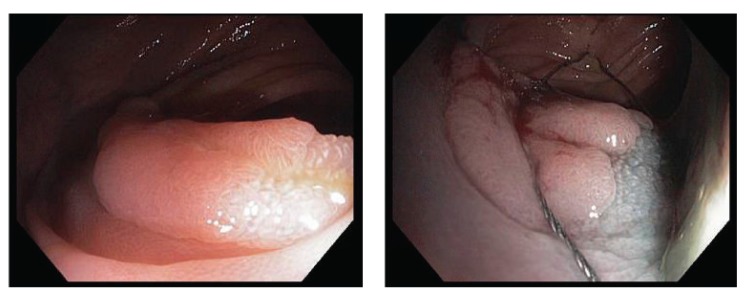
Large sessile polyp removal using Exacto cold snare.

This is a single-center, retrospective study of consecutive patients at Yale-New Haven Hospital who had CSP for large sessile colonic polyps (size ≥ 10 mm) between January 2012 and October 2013. The study was approved by the Yale University Institutional Review Board. Patients were identified through a search of electronic medical records. All cases were performed by a single attending endoscopist in consecutive patients. All procedures utilized a 9 mm braided snare designed for cold cutting (Exacto cold snare, US Endoscopy, Ohio, United States) (See Figure 1). Polyps were removed in a piecemeal fashion following submucosal saline injection with indigo carmine. Tattooing with purified carbon dye in the fold just distal to the polypectomy site to facilitate surveillance was performed in patients in whom postresection colonoscopy evaluation within 6 months to ensure complete removal was required. Data evaluated included patient age, gender, polyp size, location, pathology, the use of hemostatic techniques, and residual polyp at follow-up colonoscopy (within 6 months).

The primary outcome measure was completeness of the polypectomy and secondary outcome measures were immediate and delayed bleeding, perforation, postpolypectomy syndrome, and complication requiring admission.

## 3. Results

30 sessile polyps, at least 10 mm in size, were identified in a total of 30 patients. The mean age was 64.6 years (SD 11.01) (range 45–85). The majority of the polyps were located in the right colon with tubular adenoma or sessile serrated adenoma on histology (see Table 1). 17% of polyps were greater than 30 mm (see Table 2).

APC (Argon Plasma Coagulation) and hemostatic clips were empirically utilized in some procedures to treat the polypectomy borders or slightly oozing sites in the polypectomy base and to close the mucosal defect. In most cases, APC alone was used to treat the residual tissues at the polypectomy border (Table 3). In no cases were hemostatic techniques required for immediate active bleeding. The polyp was retrieved for pathology in all procedures (100%).

Other complications such as delayed bleeding, postpolypectomy syndrome, perforation, and any other complication requiring hospital admission were not identified in any cases.

A total of 27 patients (90%) had follow-up colonoscopy within 6 months. Among these patients 80% had complete polyp resection and did not require any further intervention. Resection was completed in the remaining patients at the time of the follow-up procedure with removal of small residual tissue, typically with biopsy. When there was no residual tissue endoscopically identified with high definition white light and digital chromoendoscopy, routine biopsies at the polypectomy site were not performed during the follow-up colonoscopy.

## 4. Discussion

We report a retrospective analysis of CSP outcomes in consecutive patients with advanced sessile polyps at least 10 mm in size. Our findings indicate that CSP was feasible in large sessile polyps with no adverse events (0/30) and with an acceptable rate of residual polyp on follow-up colonoscopy. Pohl et al. identified residual polyp in up to 31% of sessile serrated adenomas resected [11]. In 20% of our cases, small residual tissue was resected at six-month follow-up colonoscopy.

Prior studies in polyps less than 10 and 5 mm have shown a low complication rate and high degree of polypectomy completion with CSP [8, 9, 12]. Advanced sessile polyps may be difficult to remove completely using traditional methods of hot snare polypectomy due to difficulty in capturing/grasping sessile lesions, particularly in the right colon. Submucosal saline injection may further exacerbate the difficulty in grasping polyps.

A 9 mm thin wire cold snare was utilized in this case series. The thin wire and the hexagonal shape assist in facilitating polyp grasping. Cutting of flat, sessile polypoid tissue is achieved with mechanical closure of the snare. Despite the absence of cautery we did not experience active, immediate bleeding. Sessile serrated polyps have been noted to have decreased vascularity [13]. Small, focally oozing areas were typically self-limited and treated with APC. Additional study is required to determine if treatment of these areas is required and what impact hemoclip closure of the mucosal defect has on delayed bleeding in the setting of CSP. Some investigators have reported equally good results without the use of focal APC [14]. The absence of cautery with CSP prevents thermal wall injury and postpolypectomy syndrome. This could be hypothesized to be associated with a decreased risk of delayed perforation and delayed bleeding and additional study in large, randomized patient groups is warranted. In addition, the necessity of submucosal saline injection with cold snare technique has been questioned [14]. We continue to favor submucosal saline with indigo carmine injection to assist in delineating the polyp borders and to prevent injury to the muscularis propria. Further study of the impact of CSP on the muscularis propria in the right colon is warranted.

We recognize limitations of our study to include a retrospective, uncontrolled case series with a small sample size, performed by a single operator. Our results should be assessed in the context of these limitations which could underestimate the rate of complications.

We believe, however, that this technique holds promise to facilitate the complete resection of advanced sessile right colon polyps and can be performed by any experienced endoscopist [15].

## 5. Conclusion

CSP appears to be a safe and effective technique for resection of large sessile colonic polyps ≥10 mm, with an acceptable rate of residual polyp at six-month follow-up colonoscopy. Further prospective study in larger patient groups is warranted to determine optimal CSP techniques and whether CSP for large polyps has favorable efficacy in regard to complete polypectomy, procedure time, and complication rates relative to polypectomy with cautery.

## Figures and Tables

**Table 1:** Baseline characteristics.

Characteristic	
Mean age	64.60 (±11.01)
Female (*n*)	19 (63.33%)
Male	11 (36.67%)
Mean polyp size (mm)	19.00 (±8.8)
Mean procedure time (min)	57.37 (17.13)
Number of polyps in an individual patient	
(i) 1 polyp	23 (76.67%)
(ii) 2 polyps	6 (20%)
(iii) 3 polyps	1 (3.33%)
Fragmented (removed piecemeal) (*n*)	28/30 (93.3%)
Location of polyp	
(i) Right colon	24/30 (80%)
Pathology	
(i) Tubular adenoma	19/30 (63.33%)
(ii) Sessile serrated	10/30 (33.33%)
(iii) Hyperplastic	1/30 (3.33%)

**Table 2:** Polyp size.

Polyp size	Frequency	Percent %
>30 mm	5	17
20 to 30	10	33
15 to 20	4	13
10 to 15	11	37

**Table 3:** Residual tissue treatment.

Residual tissue	Frequency *n* = 30	Percent %
Intervention	21	70
APC alone	13	43
Hemoclips alone	3	10
APC & hemoclips	5	16
